# Archaeal methanogenesis in a deep terrestrial aquifer driven by *Methanothermobacter tomskensis* sp. nov.

**DOI:** 10.3389/fmicb.2026.1973590

**Published:** 2026-10-15

**Authors:** Vladimir E. Trubitsyn, Igor I. Rusanov, Viktoria I. Rechkina, Vitaly V. Kadnikov, Daria N. Zasko, Alexey V. Beletsky, Nikolai V. Pimenov, Nikolai V. Ravin, Viktoria A. Shcherbakova, Olga V. Karnachuk

**Affiliations:** 1 Skryabin Institute of Biochemistry and Physiology of Microorganisms, Federal Research Center Pushchino Center for Biological Research of the Russian Academy of Sciences, Pushchino, Moscow, Russia; 2 Laboratory of Biochemistry and Molecular Biology, Tomsk State University, Tomsk, Russia; 3 Winogradsky Institute of Microbiology, Research Centre of Biotechnology of the Russian Academy of Sciences, Moscow, Russia; 4 Institute of Bioengineering, Research Centre of Biotechnology of the Russian Academy of Sciences, Moscow, Russia; 5 Shirshov Institute of Oceanology, Russian Academy of Sciences, Moscow, Russia

**Keywords:** archaea, deep terrestrial subsurface, methanogenesis, *Methanothermobacter*, thermophiles

## Abstract

Methanogenic archaea, capable of producing organic matter from hydrogen and CO_2_ emanating from the mantle, constitute one of the key functional groups of microorganisms in the deep subsurface. Although the diversity of methanogenic inhabitants of the deep biosphere has been studied in detail, the activity of methanogens in these biotopes remains virtually uncharacterized. We have measured the rate of hyrogenotrophic methanogenesis in water from the model borehole 3P (2.7 km deep), located near the small town of Parabel in the Tomsk Region. The potential rate of hydrogenotrophic methanogenesis, measured using ^14^C-labeled sodium bicarbonate, was 5.45 ± 0.528 nmol CH_4_/dm^3^/day (0.00545 ± 0.0005 nmol CH_4_/cm^3^/day) - a value compatible to the methanogenic archaeal activity known for anaerobic sediments of surface water bodies. The 16S rRNA gene profiles of water from well 3P were dominated by s single phylotype assigned to the genus *Methanothermobacter*; it accounted for 25.54% of the total microbial community. A MAG (metagenome assembled genome) designated 3P-20-051 and belonging to the genus *Methanothermobacter* was assembled from the water metagenome of well 3P. According to GTDB taxonomy, this MAG represents a novel species within the genus. The closest validly described relative of MAG 3P-20-001 was *Methanothermobacter marburgensis* with an ANI of 92.66%. Following the characterization of this MAG, a strain of methanogenic archaea designated as K4^T^ was isolated in pure culture. Morphological, physiological, genotypic, and phylogenetic data indicate that this strain represents a new species of the genus *Methanothermobacter,* for which we propose the name *Methanothermobacter tomskensis* sp. nov. Comparison of the strain K4^T^ genome with MAG 3P-20-051 showed that they were nearly identical: the ANI value was 99.96%. Thus, *M. tomskensis* sp. nov. indeed, represents a dominant methanogenic lineage revealed by metagenomics. Strain K4^T^ is characterized by an obligately hydrogenotrophic metabolism and possibly represents the main producer of organic matter in the deep horizons of the thermal aquifer.

## 1 Introduction

Since the time when Thomas Gold hypothesized the existence of a deep biosphere (Gold, 1992), methanogenic archaea have been considered key players driving methane formation and biomass production in the deep subsurface. Methanogenic archaea have been reported to be a dominant component of microbial communities in deep (<500 m below the surface) terrestrial environments (Chapelle et al., 2002; Nyyssönen et al., 2014; Kietäväinen and Purkamo, 2015; Magnabosco et al., 2016; Magnabosco et al., 2018; Rasmussen et al., 2024). Methanogens are regularly detected in most subsurface samples analyzed, with representatives of the orders *Methanobacteriales*, *Methanomicrobiales*, and *Methanosarcinales* being more common in deep terrestrial biotopes (reviewed in Escudero et al., 2018). The main challenges to studying microbial communities in deep subsurface environments are poor accessibility, high cost of dedicated drilling projects, and potential contamination by surface microbiota during drilling. That is why much of our knowledge of microbial processes in the deep subsurface comes from industrial nuclear waste repositories, as well as from H_2_ and CO_2_ storage facilities and deep mines. Active methanogenic enrichments were obtained from underground CO_2_ storage (Jia et al., 2023). Using 16S rRNA profiling, abundant and diverse methanogenic archaea, including representatives of the genera *Methanobacterium*, *Methanosarcina,* and the family *Methanomassiliicoccaceae*, were detected in the Opalinus Clay rock formation nuclear waste repository, Switzerland (Mitzscherling et al., 2023). Methanogenic archaea of the genera *Methanobacterium* and *Methanolinea* were enriched from samples collected in the Koyna Pilot Borehole Observatory (3 km depth) in India (Mandal et al., 2022).

An alternative way to reach deep subsurface horizons is through boreholes drilled for mineral exploration purposes. These boreholes sometimes broach water aquifers and serve as a kind of “window” into the deep biosphere. In the case of an artesian aquifer, the absence of contamination by microorganisms from the surface is ensured by the nature of water flowing out of the well under pressure. One such well, designated 3P and located on the banks of the Ob River near the small town of Parabel in the Tomsk region, has become a model biotope for studying the microbial community inhabiting an aquifer nearly 3 km deep in the West Siberian Basin, the world’s largest sedimentary basin (Frank et al., 2016). Different resources estimate the well’s depth to be between 2,609 to 2,775 m (Frank et al., 2016). It passes through Quaternary, Paleogene, Cretaceous and Jurassic sedimentary sequences and reaches the Paleozoic basement, comprising by monazite and granite (Ulmishek, 2003). Numerous novel organotrophic bacteria were isolated from the water of borehole 3P, including polysaccharide-degrading *Mobilitalea sibirica* (Podosokorskaya et al., 2014), cellulolytic *Melioribacter roseus*, (Podosokorskaya et al., 2013a) and *Ornatilinea apprima* (Podosokorskaya et al., 2013b). All of these bacteria are chemoorganoheterotrophs and presumably rely on decomposition of organic matter produced by chemolithotrophes or fossilized organic remains buried in the host rocks. The 16S rRNA profiling of the 3P water showed that the majority of the microbial community consists of chemolithoautotrophes, sulfate-reducing *Desulfovirgula* and methanogenic *Methanothermobacter* (Frank et al., 2016). These results are consistent with the classical view that the predominant electron donor in deep crystalline rocks is molecular hydrogen, consumed primarily by chemolithoautrotrophic microbial communities (Nealson et al., 2005). Most of the hydrogen in the deep subsurface is thought to be formed by abiotic processes such as water radiolysis, serpentinization, graphitization, cataclasis, and degassing from the deep mantle (Gregory et al., 2019). Potentially, hydrogenotrophic methane formation is the main pathway of archaeal methanogenesis due to the availability of molecular hydrogen and may play a decisive role in autotrophic CO_2_ fixation in the deep subsurface. Bioinformatic analysis revealed that acetyl CoA-synthase, used by methanogens to fix CO_2_, is enriched in subsurface microbial communities compared to those found in surface environments (Colman et al., 2017, 2025). The authors showed that in subsurface biotopes, the number of copies of [FeFe]- and [NiFe]-hydrogeneses per megabase pair of sequence is an order of magnitude higher compared to those in surface environments. To resolve the discrepancy between metagenomic results for water from borehole 3P – which indicate a predominance of chemolithotrophic methanogens - and cultivation results – which yield chemoorganotrophs – we aimed in this study to isolate the dominant hydrogenotrophic methanogen and supplement this with measurements of methane production using a radioactive tracer technique. Obtaining the genome of the dominant methanogen from metagenomic data preceded the isolation of a pure culture.

## 2 Materials and methods

### 2.1 Deep water sampling, chemical analyses, direct cell count and DNA isolation

Water samples were collected in February 2020 directly from the discharge at the wellhead of borehole 3P. Borehole 3P is located on the western bank of the Ob River, between the small towns of Parabel and Narim in the Tomsk region, Western Siberia, Russia. Water temperature, pH and Eh values were determined on-site using an HI 8314 pH meter (Hanna Instruments, Germany) with appropriate electrodes. Water samples for ion and elemental analysis were filtered (0.45 or 0.22 μm) using Millex-GS filter units (Merk Millipore) and stored in polyethylene or glass flasks in a refrigerator at 4 °C. Ion concentrations were measured using ion chromatography (Dionex, CA, USA). The elemental composition of the water was analyzed by ICP-MS (Plasma Chemical-Analytical Centre, Tomsk). Gas content was determined using a Kristall-5000.1 gas chromatograph (Russia) equipped with a thermal conductivity detector (katharometer). The carbon isotopic composition of methane (δ^13^C-CH_4_), was measured using a TRACE GC gas chromatograph (Thermo Fisher Scientific, Germany) connected to a Delta Plus mass spectrometer at the Federal Research Centre of Biotechnology of the Russian Academy of Sciences, as previously described (Savvichev et al., 2023).

For direct cell counting, water samples were fixed with glutaraldehyde (final concentration 2%), filtered through black polycarbonate membranes (Millipore), and stained with acridine orange. Cell counting on filters was performed using an epifluorescence microscope (Axio Imager D1, Carl Zeiss, Oberkochen, Germany) equipped with a visualization system (Image Scope Color, Leica Biosystems, Nussloch, Germany) at ×1,000 magnification.

Microbial biomass from 50 L of water was collected by filtration through 0.22 μm cellulose nitrate membranes (Sartorius, Germany). The filters were frozen in liquid nitrogen and then ground and melted with TE buffer in a water bath at 37 °C. Total DNA was extracted using the Power Soil DNA isolation kit (MO BIO Laboratories, Carlsbad, CA, United States). Approximately 1.5 μg of DNA was obtained.

### 2.2 Measurement of potential hydrogenotrophic methanogenesis and microbial sulfate-reduction rates with a radioactive tracer

The potential rate of hydrogenotrophic methanogenesis was determined using ^14^C-labeled bicarbonate (NaH^14^CO_3_ specific activity 2.04 GBq mmol^−1^, Amersham, UK). The sulfate reduction rate (SRR) was measured using ^35^S-sulfate (Na_2_^35^SO_4_, specific activity 38.8–59.2 TBq/mmol, PerkinElmer, Waltham, Massachusetts USA). Water samples were placed in sterile 30 mL penicillin vials leaving no headspace and tightly sealed with butyl rubber stoppers directly at the wellhead. Aliquots (200 mL) of NaH^14^CO_3_ (5.0 μCi) and Na_2_^35^SO_4_ (3 μCi) were injected through a butyl rubber stopper. The vials were incubated in the dark at an *in situ* temperature of 55–57 °C, for 24 h, followed by the addition of 1 mL of 0.1 M KOH to stop microbial activity and fix the sulfide. Subsequent analytical procedures for measuring ^14^CH_4_ and reduced ^35^S species formed in the samples were performed under laboratory as previously described (Pimenov and Bonch-Osmolovskaya, 2006; Karnachuk et al., 2023). The ^35^S-tracer was measured in the acid volatile sulfide fraction (AVS) and the chromium-reducible sulfur fraction (CRS) as previously described (Karnachuk et al., 2023). The radioactivity (^14^C and ^35^S) of methane and reduced sulfur species was measured using a Packard TRI-CarbTR 2,400 (PerkinElmer, Shelton, USA) liquid scintillation analyzer. Rates of potential hydrogenotrophic methanogenesis and sulfate reduction were calculated using the mean values of three replicate measurements for each sample.

### 2.3 16S rRNA gene profiling, sequencing of metagenomic DNA, and assembly of MAGs

Amplification of the 16S rRNA gene fragments covering the V3–V4 variable regions was performed using universal primers 341F_Fr (5′-CCT AYG GGD BGC WSC AG-3′) and 806R_Fr (5′-GGA CTA CNV GGG THT CTA AT-3′). The resulting PCR fragments were sequenced on the Illumina MiSeq instrument in paired-end mode (2 × 300 bp). Paired overlapping reads were merged using FLASH v.1.2.11 (Magoč and Salzberg, 2011). The resulting 34,470 16S rRNA gene sequences were clustered into operational taxonomic units (OTUs) at 97% similarity threshold using USEARCH v.11 (Edgar, 2010). Taxonomic identification of OTUs was performed by comparison with the SILVA v.138.1 database (Quast et al., 2013) using the VSEARCH v. 2.28.1 (Rognes et al., 2016). To estimate the relative abundance of OTUs, all reads were mapped to OTU sequences at a global identity threshold of 97% using USEARCH.

Metagenomic DNA was sequenced using Illumina and Oxford Nanopore technologies. For Illumina sequencing, the library was prepared using NEBNext Ultra II DNA Library prep kit (New England Biolabs, Ipswich, MA, USA) and sequenced on an Illumina HiSeq 2,500 instrument in paired-end read format (2 × 150 nt). A total of 80,514,285 read pairs (~ 24 Gb) was generated. Paired reads were merged using FLASH v.1.2.11 (Magoč and Salzberg, 2011). Adapter removal and trimming of low quality read ends were performed using Cutadapt v.1.8.3 (Martin, 2011) and Sickle v.1.33 (https://github.com/najoshi/sickle), respectively.

Genomic DNA was additionally sequenced on a MinION instrument (Oxford Nanopore Technologies, Oxford, UK) using the 1D Genomic DNA ligation sequencing kit LSK-109 and FLO-MIN106D cell. A total of 4.5 Gb (1,889,561 reads with an N50 length of 4,549 nt) were obtained. The MinION reads were assembled using Flye v. 2.8.1 (Kolmogorov et al., 2019). The sequences of the assembled contigs were polished with Illumina reads using two iterations of Pilon v.1.24 (Walker et al., 2014).

Contigs were binned into MAGs using MetaBAT2 v.2.15 (Kang et al., 2019), the completeness and contamination of obtained MAGs was assessed using CheckM2 (Chklovski et al., 2023). The assembled MAGs were taxonomically assigned using the GTDB-Tk v. 2.7.1 tool (Chaumeil et al., 2022) and Genome Taxonomy Database (GTDB) v.232 (Parks et al., 2018).

### 2.4 Pure culture isolation and characterization

To obtain a methanogenic enrichment, we added 20% (v/v) of the well water to the anaerobic (Hungate, 1969) MG medium of the following composition (per liter of distilled water): KH_2_PO_4_ 0.5 g, MgSO_4_ × 7H_2_O 0.4 g, NaCl 0.4 g, NH_4_Cl 0.4 g, CaCl_2_ × 2H_2_O 0.05 g, NaHCO_3_ 4.0 g, cysteine-HCl 0.05 g, Na_2_S × 9H_2_O 0.05 g, 2 mL of 0.1% FeSO_4_ × 7H_2_O in 0.1 N H_2_SO_4_, 1 mL of SL-10 trace element solution (DSMZ medium 320), 1 mL of 0.2% resazurin, and 10 mL of Wolin’s vitamin solution (DSMZ medium 318). Sodium acetate (30 mM) and sodium formate (30 mM) were added as substrates, and the flasks were filled with a gas mixture of H_2_ + CO_2_ (4,1) to a pressure of 1 atm. We incubated the enrichments at 37 and 55 °C and measured methane production monthly using gas chromatography. After 12 months of incubation, methane appeared in the headspace of the flask incubated at 55 °C. After the methane concentration reached 40%, we transferred the entire content of the flask with active methanogenesis into Hungate tubes supplied with 5 mL of MG medium and ampicillin (0.5 mg/mL). A pure culture of the methanogenic strain was obtained using the method of serial tenfold dilution, with the sequential addition of antibiotics (vancomycin 0.5 mg/mL, methicillin 1 mg/mL, and erythromycin 0.025 mg/mL) to the same medium and the use of H_2_/CO_2_ gas mixture as the sole substrate. We designated this novel archaeon as strain K4^T^. To determine the purity of the resulting culture, we used diluted trypticase soy broth (¼-strength TSB, Difco) and MG medium supplemented with a mixture of glucose (10%) and peptone (10%) in an amount of 0.1 mL per 10 mL of medium. Visual microscopic observation verified the purity of the culture.

We performed direct observations of the strain cells in phase-contrast mode using an Axiostar PLUS microscope (Carl Zeiss, Germany) at 1000 × magnification. The Gram staining method was used for the differential staining of cells. We prepared ultrathin sections of cells using a modified Reynolds technique as described previously (Shcherbakova et al., 2005) and captured micrographs using a JEM 100 transmission electron microscope (JEOL, Japan).

We tested the following substances and mixtures as potential substrates for the growth of strain K4_T_: H_2_/CO_2_, formate, methanol + H₂, ethanol + CO_2_, isobutanol + CO_2_, isopropanol + CO_2_, acetate, acetate + H_2_, methanol, and methylamine. Gases and gas mixtures were sparged into the samples to a pressure of 1 atm, while liquid substrates were added to a final concentration of 0.1% (v/v). Bicarbonate or phosphate buffers maintained a neutral pH. We studied the effect of temperature on the growth of strain K4_T_ by culturing it at 37, 45, 50, 55, 60, 70, and 75 °C. We determined the pH optimum in the range from 6.0 to 7.9 using citrate and phosphate buffer systems, as well as HEPES and EPPS. To evaluate the effect of sodium chloride concentrations on the growth of the strain, we added 0, 9, 17, 86, 171, 257, 342, 513, and 600 mM NaCl to the medium. To study the stimulating effect, we supplemented the medium containing the main substrate (H_2_/CO_2_ mixture) with formate, acetate, casamino acids, yeast extract, coenzyme M (CoM), or trypticase peptone to a final concentration of 0.1% (v/v). We analyzed the effect of rumen fluid at a concentration of 20 mL/L. We determined the antibiotic resistance of strain K4_T_ using (mg/ml): methicillin (1), vancomycin (0.5), streptomycin (0.2), kanamycin (0.1), erythromycin (0.05), tetracycline (0.01), bacitracin (0.005), and amikacin (0.001).

Cell susceptibility to lysis was assessed in several ways: exposure of the culture to a 1% (v/v) SDS solution for 10 min, centrifugation at 12,000 × g for 5 min, and placing the cells under hypotonic conditions. We observed the experimental results visually under a microscope.

We monitored the growth of strain K4^T^ by the methane content in the gas phase using a Pye Unicam 304 gas chromatograph (UK) with a flame ionization detector on a glass column (length 1 m, diameter 2 mm) filled with Porapak Q, 80–100 mesh (Fluka, Germany). The column, injector, and detector operated at 110, 120, and 170 °C, respectively. The carrier gas was CO_2_ with a flow rate of 20 mL per min. The sample volume for methane measurements was 0.2 mL.

Isolation and purification of chromosomal DNA was carried out using a modified Marmur method (Marmur, 1961). For amplification of the target genes we used a Tertsik amplifier (DNA-Technology, Russia). The total volume of the reaction mixture was 25 μL and included the following components: 1x Taq polymerase buffer (Fermentas, Lithuania); 10 ng DNA template; 50 μM of each dNTP (Fermentas, Lithuania); 0.25 μM of each primer. Universal archaeal primers 21f (5’-TTCCGGTTGATCCYGCCGGA-3′) and 1378r (5’-TGTGCAAGGAGCAGGGAC-3′) were used to amplify fragments of the 16S rRNA gene. Primers ME-1 (5’-GCMATGCARATHGGWATGTC-3′) and METH-r (5’-YTGDGAWCCWCCRAAGTG-3′) were used to amplify fragments of the mcrA gene.

Genomic DNA preparation and whole-genome sequencing were performed at BioSpark (Troitsk, Russia). Genomic DNA was isolated using the FastDNA SPIN Kit (MP Biomedicals, USA) by column precipitation on silica gel. Read libraries were prepared using KAPA HyperPlus Kits (Kapa Biosystems, USA) according to the manufacturer’s recommendations. The fragment composition of the libraries was verified by capillary electrophoresis on a QIAxcel Advanced System using the QIAxcel DNA High Resolution Kit (Qiagen, Germany) and normalized by quantitative PCR on a CFX Connect Real-Time PCR System (Bio-Rad, USA). Paired-end sequencing of 101 bp read libraries was performed on an Illumina NovaSeq 6000 platform (Illumina, USA).

Read quality was assessed using FastQC v.0.11.9. Read libraries were edited using Trimmomatic v.0.36 (Bolger et al., 2014) with adapters removed (from the TruSeq3-PE-2 set). We edited read libraries using Trimmomatic v.0.36 (Bolger et al., 2014) with adapters removed (from the TruSeq3-PE-2 set). The SPAdes v.3.15.3 (Bankevich et al., 2012) assembled the genome using standard settings. CheckM v.1.0.18 (Parks et al., 2015) assessed genome completeness. We deposited the resulting genome in NCBI (RefSeq GCF_022014235.1) and saved the read libraries under accession number SRS11832015. To confirm the phylogenetic position and search for the closest related strains, we used the GTDB-Tk v.2.3.2 program (Chaumeil et al., 2022).

The ANI and AAI coefficients between the studied strain and its closest relatives were calculated using the GET_HOMOLOGUES v. 22082022 program (Contreras-Moreira and Vinuesa, 2013), while digital DDH (dDDH) values were determined via the GGDC web service (https://ggdc.dsmz.de/). Phylogenetic trees based on the aligned sequences of 49 housekeeping genes were constructed using SpeciesTreeBuilder v. 0.1.4 (https://github.com/kbaseapps/SpeciesTreeBuilder) within the KBase platform (Arkin et al., 2018). This involved sequence trimming with the Gblocks algorithm and tree reconstruction via the approximate maximum-likelihood method (Price et al., 2010). Homologous genes were identified by comparing the amino acid sequences of the studied strain against other representatives of the genus *Methanothermobacter* using psiblast v. 2.12.0 from the NCBI BLAST+ software suite (Camacho et al., 2009). The blast results were subsequently filtered using a custom Python 3 script (https://github.com/TVApro/FindMoreGenes) with an E-value threshold of 1e-70 and a minimum query coverage of 70%.

## 3 Results

### 3.1 Physicochemical characteristics of the thermal water from the 3P borehole and potential hydrogenotrophic methanogenesis

Detailed water chemistry for well 3P was reported in our previous study (Frank et al., 2016). In February 2020, the temperature measured at the wellhead - 57 °C - exceeded previously measured values (Table 1). The water was characterized by reducing conditions (Eh = −270 mV) and a circum-neutral pH, which is consistent with the results of our previous measurements conducted between 2009 and 2013 (Frank et al., 2016). Methane was the major constituent of the gas phase, accounting for nearly 70%, and its concentration reached a significant level of 678 μmol/L (Table 2). Molecular nitrogen was the second constituent with the share of 27.5%, followed by oxygen, which made up 4.5% of the gas phase. Molecular hydrogen accounted for a negligible fraction - 0.0046%. The carbon isotopic composition of methane was −54.9 ‰. The δ^13^C value of dissolved organic carbon was −29.3 ‰. The total cell number, determined by direct counting method with acridine orange, was low, amounting to 0.856 ± 0.372 × 10^3^ cells/mL.

**Table 1:** Physicochemical characteristics of water from borehole 3P, determined at different sampling times (* data from Frank et al., 2016, with additions; ** not determined).

Parameter	2009 August*	2010 February*	2010 August*	2013 August*	2020 February
T ^o^C	48.9	50.2	45.2	50.0	57.0
pH	7.5	7.4	6.9	7.3	7.42
Eh, mV	−248	−299	−284	−283	−270
SO_4_^2−^, mg/L	4.2	<2.0	4.23	25.0	2.1
Cl^−^, mg/L	8,875	9,198	nd**	nd**	6,931
NO_3_^−^, mg/L	0.5	<0.1	nd**	nd**	<0.1
Br^−^, mg/L	29.92	32.14	nd**	nd**	1.0
Na^+^, mg/L	4,280	4,950	4,320	4,339	4,250
K^+^, mg/L	30.19	33.2	31.2	37.4	1.0
Mg^2+^, mg/L	5.64	<1.2	18.0	5.4	1.5
Ca^2+^, mg/L	1,375	1,242	1,240	1,166	1,200

**Table 2:** Characteristics of water from borehole 3P related to the methane cycle, measured in February 2020.

Parameter	Value
CH_4_ share in gas phase, %	68.2
N_2_ share in gas phase, %	27.5
O_2_ share in gas phase, %	4.5
H_2_ share in gas phase, %	0.0046
CO_2_ share in gas phase, %	0.170
CH_4_ content, μL/L (μmol dm^−3^)	15,188 (678)
δ^13^C-CH_4_, ‰	−54.94
δ^13^C-DIC, ‰	−29.27
Total microbial cell count, cells/mL	0.856 ± 0.372 ^.^10^3^
Hydrogenotrophic methanogenesis rate, nmol CH_4_/dm^3^/day (nmol CH_4_/cm^3^/day)	5.45 ± 0.528 (0.00545 ± 0.000528)

The potential rate of hydrogenotrophic methanogenesis measured using a radioactive tracer (^14^C-bicarbonate) at *in situ* temperature in 3P water, was relatively high reaching 5.45 ± 0.528 nmol CH_4_ dm^−3^ day^−1^ (0.00545 ± 0.000528 nmol CH_4_ cm^−3^ day^−1^) (Table 2). The rate of sulfate reduction under *in situ* conditions was 2.61 ± 0.00003 nmol SO_4_^2−^ dm^−3^ day^−1^ (0.00261 ± 0.000000003 nmol SO_4_/cm^3^/day).

### 3.2 Analysis of microbial community composition using 16S rRNA gene profiling

The composition of microbial community in the 3P water sample was characterized by 16S rRNA gene profiling. Archaea accounted for 25.8% of the total 16S rRNA gene sequences and were dominated by a single phylotype assigned to the genus *Methanothermobacter* (25.54% of the total), comprising hydrogenotrophic methanogens. Other methanogens were found in minor amounts: *Methanobacterium* (0.003%), *Methanosaeta* (0.01%), and methylotrophic methanogens of the family *Methanomassiliicoccaceae* (0.22%).

The bacterial community was more diverse and was dominated by the phyla *Firmicutes* (*Bacillota*, 34.7%), *Proteobacteria* (*Pseudomonadota*) of the classes alpha (7.4%) and gamma (18.6%), *Chloroflexota* (5.5%), and *Actinobacteriota* (3.2%); the shares of each of the other phyla were less than 1%. Among known sulfate reducers, *Firmicutes* of the genus *Desulfovirgula* (Kaksonen et al., 2007) predominated (17.9%), while other lineages were found in the minority (*Desulfofundulus* - 0.22%, *Desulforudis* - 0.01%). The relative abundance of known methanotrophs was very low. Only single 16S rRNA gene sequences (out of a total of 34,470) were assigned to the genera *Methylocystis, Methylotenera, Methyloversatilis, Methylococcus*, and *Methylomonas*.

### 3.3 The complete genome of *Methanothermobacter* sp., assembled from the metagenome of borehole 3P water

To obtain the genomes of microbial community members, metagenomic sequences obtained using Illumina and Oxford Nanopore technologies were assembled into contigs, which were then binned into MAGs. Nine MAGs with more than 90% completeness and less than 5% contamination were obtained. One MAG (designated 3P-20-001, GenBank CM185573) consisted of a single circular contig of 1,745,437 bp in length. CheckM2 estimates that this MAG is 99.78% complete with a possible contamination level of 0.02%. Taxonomic assignment of this MAG in the GTDB taxonomy placed it into the genus *Methanothermobacter*, where it represented a novel species. The closest relative of 3P-20-001 archaeon appeared to be *Methanothermobacter* sp. KEPCO 2 (GCF_041228365.1) with 92.75% ANI. Considering validly described species, the closest microorganism was *Methanothermobacter marburgensis* str. Marburg (GCF_000145295.1) with an ANI of 92.66%, which is below the 95% threshold for species delimitation (Jain et al., 2018). Therefore, strain 3P-20-001 likely represented a novel species of *Methanothermobacter*.

### 3.4 *Methanothermobacter tomskensis* sp. nov. isolation and characterization

The finding of *Methanothermobacter* among the dominant members of the microbial community prompted us to isolate this archaeon in pure culture. Following prolonged cultivation of a thermophilic enrichment culture and repeated serial transfers using a gas mixture (H₂ + CO₂) as the source of carbon and energy, a pure culture of a rod-shaped methanogen was isolated.

Cells of strain K4^T^ were non-motile, long, thin rods measuring 2.0–5.4 × 0.25–0.40 μm, occurring singly or in pairs, or forming short threads (Figure 1A). They stained as Gram-positive, and the presence of Gram-positive-type cell wall was confirmed by TEM micrographs of ultra-thin sections (Figure 1B). Cells were resistant to lysis under hypotonic conditions, and withstood centrifugation and exposure in SDS. The culture grew on liquid nutrient media, forming white turbid sediment.

**Figure 1:**
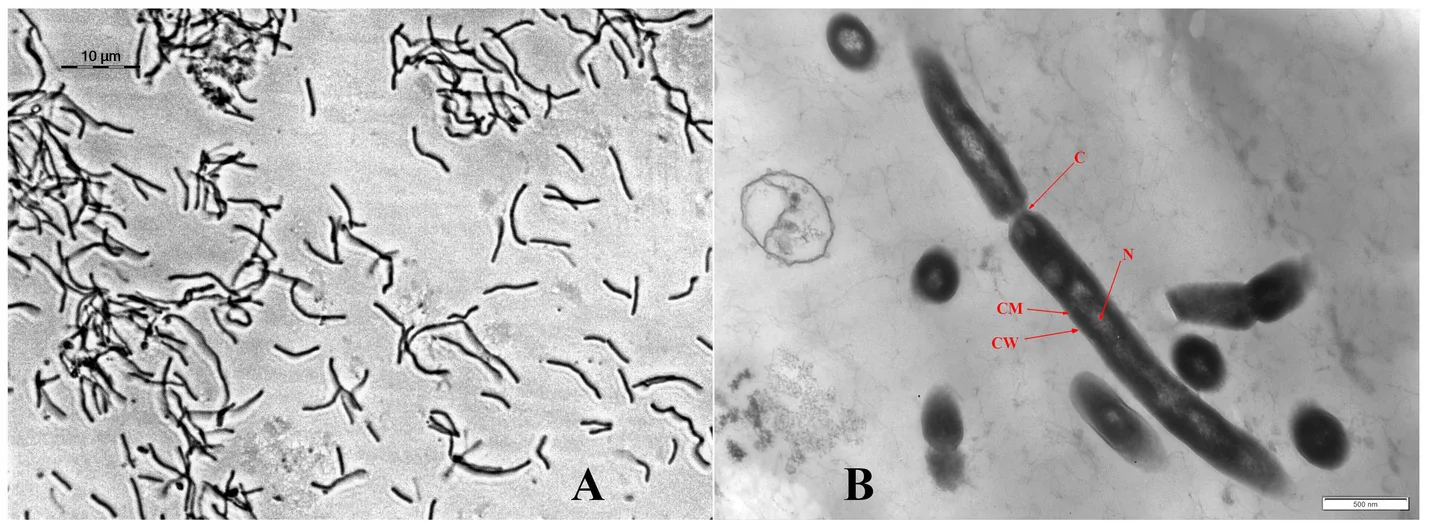
Micrographs of strain K4^T^ cells: **(A)** phase-contrast microscopy (scale bar, 10 μm); **(B)** ultrathin sections, transmission electron microscopy (scale bar, 500 nm). C, constriction between dividing cells; CW, cell wall; CM, cytoplasmic membrane; N, nucleoid.

Strain K4ᵀ utilized an H₂/CO₂ gas mixture as the sole carbon and energy source and did not grow on formate, methanol + H₂, ethanol + CO₂, isobutanol + CO₂, isopropanol + CO₂, acetate, acetate + H₂, methanol, or methylamine (Supplementary Table S1). It could grow on a mineral medium without growth factors, but the addition of yeast extract, casamino acids, coenzyme M (CoM), trypticase, or acetate slightly accelerated its growth.

The growth rate of the strain was highest at 60 °C; growth and methanogenesis were not observed at temperatures below 45 °C or above 70 °C (Figure 2A). The optimum pH for growth was 7.5, and the culture grew within the range of 6.1 to 7.9 (Figure 2B). The studied strain also grew across the entire range of NaCl concentrations tested (0–600 mM), with the optimal growth observed at 17 mM NaCl (Figure 2C). The minimum measured doubling time (t_d_) under optimal conditions was 6.5 h.

**Figure 2:**
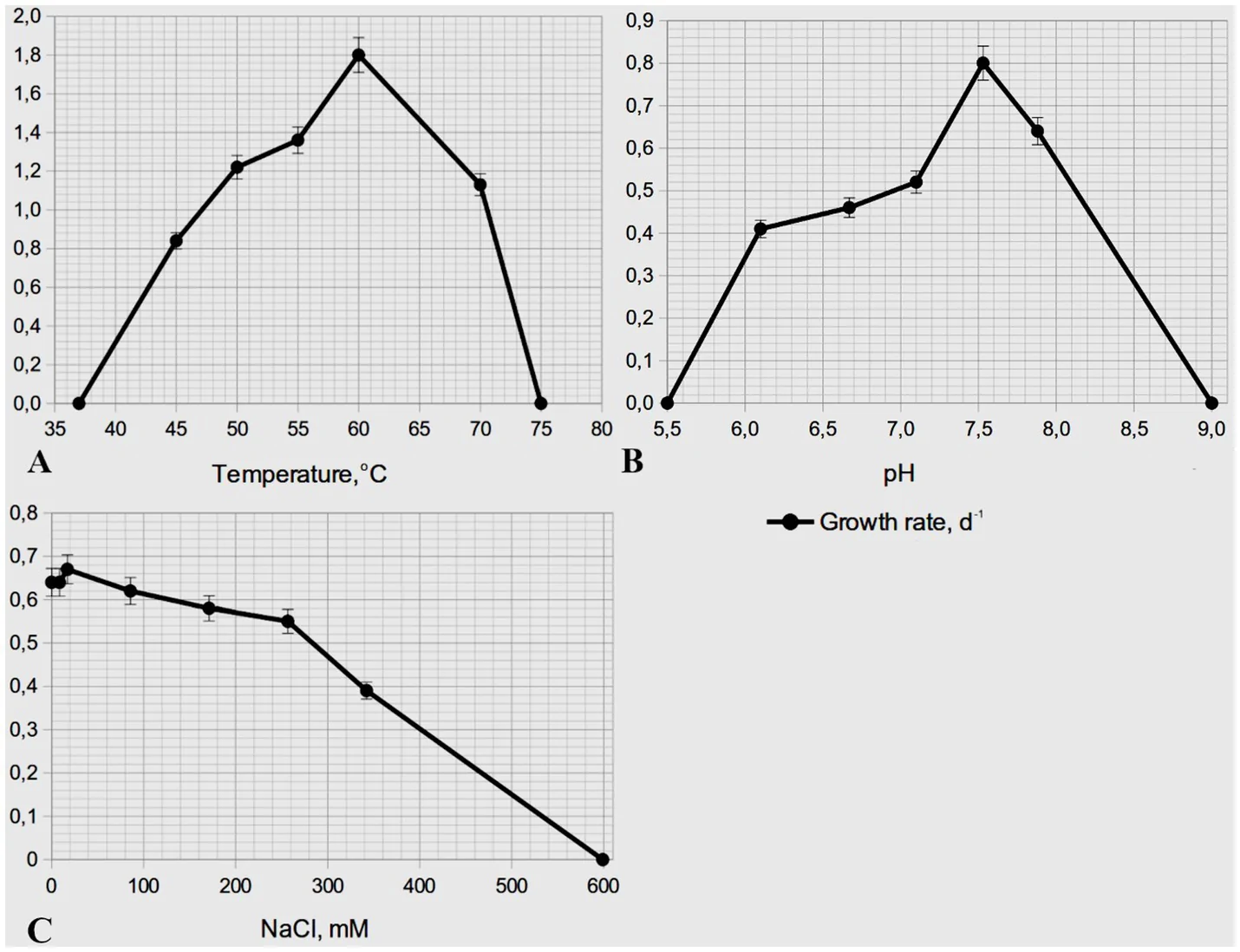
Effect of the temperature **(A)**, the pH of the medium **(B)**, and NaCl **(C)** on the growth rate of strain K4^T^.

The strain was resistant to methicillin, vancomycin, streptomycin, erythromycin, and tetracycline. Growth was partially inhibited by kanamycin and amikacin, whereas the addition of bacitracin completely inhibited it.

### 3.5 Phylogenetic and genome-based analysis

The 16S rRNA gene sequence of strain K4ᵀ showed the highest similarity (95.41–99.51%) to representatives of the genus *Methanothermobacter*, with *M. marburgensis* strain Marburgᵀ being the closest relative (99.51% similarity). Comparison of the mcrA gene sequence (encoding the alpha subunit of methyl-coenzyme M reductase, MCR) from strain K4^T^ with those from closely related species revealed a similar pattern (Supplementary Figure S1). The obtained *Methanothermobacter* sp. K4ᵀ genome assembly had a length of 1,744,648 bp and consisted of 12 scaffolds with an N50 value of 572,094 bp. The genome (RefSeq annotation) contained 1,854 genes, including 1,759 protein-coding genes. The coding coefficient was 91.26%, and the genome completeness estimated by CheckM was 98.4% with a contamination level of 0.25%.

Using GTDB-Tk, it was confirmed that the closest relative of the new strain is *M. marburgensis* Marburgᵀ (RefSeq GCF_000145295.1). The ANI, AAI, and dDDH values between the genomes of these microorganisms were 94.21, 93.75, and 48.50%, respectively, which is slightly below the species delimitation thresholds. The phylogenetic tree constructed based on the analysis of a concatenated set of housekeeping genes is topologically similar to the 16S rRNA gene tree, confirming our data (Figure 3). Comparison of the *Methanothermobacter* sp. K4ᵀ genome with MAG 3P-20-001 showed that they were nearly identical, with an ANI value of 99.96%. Therefore, the cultivated strain K4ᵀ indeed represents the dominant methanogenic lineage revealed by metagenomics.

**Figure 3:**
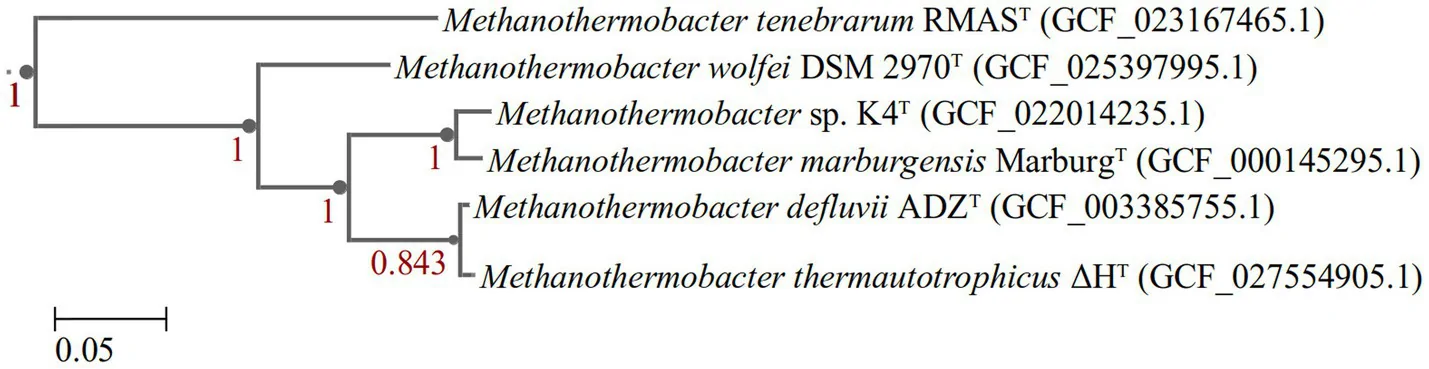
Phylogenetic tree based on the analysis of 49 housekeeping gene clusters (amino acids sequences) in the genome of strain K4^T^ and type strain genomes of *Methanothermobacter* genus available in the NCBI databases.

The K4ᵀ genome harbored all the core genes encoding the key enzymes of the methanogenesis pathway (Supplementary Table S2). Notably absent were the genes encoding the molybdenum-containing isoform of formylmethanofuran dehydrogenase (specifically, *fmdC* and *fmdB*), which is characteristic of the closest related strain *M. marburgensis* Marburgᵀ. Two copies of the gene encoding AMP-forming acetyl-CoA ligase (*acs*, EC 6.2.1.1), all genes of the CODH/ACS complex (*cdhA*, *cdhB*, *cdhC*, *cdhD*, and *cdhE*), and CO dehydrogenase maturation factor genes (such as *MTBMA_c02900* and *MTBMA_c15030*) responsible for acetate uptake and CO₂ fixation were detected. The energy-converting hydrogenase (Eha), which is also involved in CO₂ fixation, was represented by its encoding genes (*ehaA–T*) present in the genome.

According to the study by Cassini et al. (2023), the utilization of formate as the sole substrate in *M. thermoautotrophicus* strain Z-245 is enabled by proteins encoded by a specialized gene cassette, *fdhBAC* (*ISG36_RS07695*, *ISG36_RS07700*, and *ISG36_RS07705*). In contrast, the “classical” formate dehydrogenase FdhAB (*ISG36_RS05585* and *ISG36_RS05580*) is involved in the synthesis of formate from CO₂ and H₂ for anabolic processes, particularly for purine biosynthesis. No homologs of the *fdhBAC* cassette genes were detected in the genome of strain K4ᵀ; however, the genes encoding the “classical” formate dehydrogenase (*fdhAB*) were present (*L5462_RS02985* and *L5462_RS02990*). This is consistent with the observed inability of the studied strain to use formate as a sole carbon and energy source.

In addition, we identified a distinct formate dehydrogenase variant in the genome of strain Z-245, comprising an unusually large FdhA subunit (also known as FdhF) and an accessory protein FdhD (*ISG36_RS07560* and *ISG36_RS07585*). These genes are located within a cluster that also contains genes for molybdopterin cofactor biosynthesis (*moaA*, *mobB*) and respiratory chain subunits (*nuoE*, *nuoF*) (cluster NZ_CP064337.1:1424911–1,414,165). A homologous cluster was identified in strain K4ᵀ (NZ_JAKLTD010000001.1:186791–197,543). Comparative analysis revealed that this formate dehydrogenase variant (designated here as FdhFD) is more prevalent among *Methanothermobacter* species than the “classical” FdhAB (Supplementary Table S3). As the *fdhFD* cluster lacks the *fdhB* gene, which is essential for electron transfer to the F₄₂₀ cofactor, but includes genes for respiratory chain components, we propose that this enzyme variant exchanges electrons directly with the respiratory chain.

Furthermore, genes encoding pyruvate-formate lyase (Pfl), homologous to those found in *M. thermoautotrophicus* ΔHᵀ, were detected in the genome of strain K4ᵀ. Notably, these *pfl* genes were absent in the other *Methanothermobacter* genomes examined (Supplementary Table S2).

Genes responsible for the utilization of methanol and methylated derivatives (*mtt*, *mtb*, and *mtm*) were absent. The strain contains genes for the hydrogen-dependent methylenetetrahydromethanopterin dehydrogenase (*hmd*, WP_237779322.1), which is an alternative to Mtd and is present in some hydrogenotrophic methanogens. F₄₂₀-reducing (frhABDG) and F₄₂₀-non-reducing (mvhADG) hydrogenases were detected.

Recently, *M marburgensis* Marburg^T^ was shown to exhibit methanogenic growth on H₂/CO₂/CO mixtures, as well as on CO as a sole substrate (Diender et al., 2016). Strain K4^T^ possesses a complete set of genes required for CO utilization, including the carbon monoxide dehydrogenase/acetyl-CoA synthase complex: *cdhA* (L5462_RS00085), *cdhC* (L5462_RS00075), *cdhD* (L5462_RS00065), and *cdhB* (L5462_RS00080), along with the acetyl-CoA decarbonylase/synthase gene *acsC* (L5462_RS00060). Consequently, given its close phylogenetic relationship to strain Marburg^T^, strain K4^T^ is highly likely capable of carboxydotrophic growth.

The new methanogenic archaeon also differs physiologically from the closest typical organisms, including *M. marburgensis* Marburgᵀ (Table 3). Strain K4ᵀ has a lower temperature optimum, the optimal NaCl concentration for growth is almost twice as high as that of the Marburgᵀ strain, and growth-stimulating additives have a more significant effect on its growth. Pairwise comparisons of genomes showed that the differences between the K4ᵀ and Marburgᵀ strains are quite significant, confirming that the studied methanogen belongs to a new species of the genus *Methanothermobacter*. Thus, based on morphological, physiological, genotypic, and phylogenetic data, it can be concluded that the new methanogenic archaeon is a representative of a new species of the genus *Methanothermobacter*.

**Table 3:** Phenotypical features of strain K4^T^ and type strains of closely related species.

Characteristic	1	2	3	4	5
Cell sizes, μm	4.0–4.8 × 0.3–0.4 (in chains up to 18–20 μm long)	3.0–20.0×0.4	3.0–120.0×0.4	2.5×0.4	1.4–6.5×0.4
Gram staining	+	+	+	+	−
Optimum conditions (range)					
Temperature, ^o^C	60 (45–70)	65 (45–70)	65–70 (40–75)	55–65 (37–74)	57 (45–65)
pH	7.5	6.8–7.4	7.2–7.6	7.0–7.5	7.6
NaCl concentration, M	0.017	0.009	0.010	0–0.170	ND
Substrates	H	H	H	H, F	H
Growth-promoting agents	Y, А, K, C, T	−	Cys	Y	Y, C
G + C content in DNA, mol %	48.67 (Gs)	48.60 (Gs)	51.20 (HPLS), 49.50 (Gs)	61.0 ± 1.0 (Tm)	44.70 (Tm)
Isolation source	geothermal waters from the depth of ~2.8 km, Russia	mesophilic sewage sludge, Germany	anaerobic sewage sludge digester, USA	mixture of sewage sludge and river sediment, Germany	thermophilic methane tank, USSR

Strains: 1 – K4^T^, 2 – *M. marburgensis* Marburg^T^ (Wasserfallen et al., 2000), 3 – M. thermoautotrophicus ΔH^T^ (Wasserfallen et al., 2000), 4 – *M. wolfei* VKM B-1829^T^ (Wasserfallen et al., 2000), 5 – *M. thermophilus* M^T^ (Laurinavichus et al., 1988). –, negative, +, positive, ND, no data. Substrates: H, H^2^/CO^2^; F, formate. Additives: A, acetate; Y, yeast extract; K, casamino acids; T, trypticase peptone; C, coenzyme M; Cys, cysteine. Determination of G + C composition: Gs, genomic sequencing; HPLS, high-performance liquid chromatography; Tm, melting temperature.

### 3.6 Description of *Methanothermobacter tomskensis* sp. nov.

*Methanothermobacter tomskensis* (tom.sken’sis. N. L. masc. Adj. *tomskensis*, pertaining to the Tomsk region, Russia, where the type strain was isolated).

Cells are non-motile, non-spore-forming, long, thin rods with dimensions of 2.0–5.4 × 0.25–0.40 μm, occurring singly, in pairs, or forming short threads. They stain Gram-positive, are resistant to lysis under hypotonic conditions, and withstand centrifugation and exposure to 1.0% SDS. In liquid nutrient media, the culture grows forming a white sediment. Strictly anaerobic and thermophilic. The optimum temperature for cultivation is 60 °C; growth is not observed below 45 °C or above 70 °C. The optimum pH and NaCl concentration are 7.5 and 17 mM, respectively. The sole substrate utilized is a gas mixture of H₂/CO₂ (4:1, v/v). It does not use formate, acetate, methanol, methylamine, methanol + H₂, ethanol + CO₂, isobutanol + CO₂, isopropanol + CO₂, or acetate + H₂ as sole carbon and energy sources. It can grow without growth factors, but the addition of yeast extract, casamino acids, coenzyme M, trypticase, or acetate slightly accelerates growth. It is resistant to methicillin, vancomycin, streptomycin, erythromycin, and tetracycline. Kanamycin and amikacin partially inhibit growth, whereas bacitracin completely inhibits it.

The type strain, K4ᵀ (=VKM B-3410ᵀ = JCM 39440ᵀ), was isolated from the geothermal water of borehole 3P (58.83° N, 81.5° E) in the Tomsk region, Russia. The DNA G + C content of the type strain is 48.67 mol% (calculated from the genome).

## 4 Discussion

The measured rate of potential hydrogenotrophic methanogenesis in water from well 3P (5.45 ± 0.528 nmol CH_4_/dm^3^/day) was relatively high and, in terms of order of magnitude, corresponded to the mean values characteristic of anaerobic surface water environments, such as freshwater and marine bottom sediments (Savvichev et al., 2025; Savvichev et al., 2023). For example, the rate of hydrogenotrophic methanogenesis, measured using radioactive bicarbonate in sediments from marine basins of the Barents and White Seas isolated by dams, ranged from 3 to 40 nmol CH_4_/dm^3^/day (Savvichev et al., 2025). The rate of autotrophic methanogenesis in Laptev Sea shelf sediments in the area of methane seeps ranged from 0.4 to 5.0 nmol dm^−3^ day^−1^ (Savvichev et al., 2023). In the water column of the meromictic Lake Svetloe, the maximum rate of hydrogenotrophic methanogenesis, measured in the chemocline was 0.13 μmol CH_4_ L^−1^ day^−1^ (Savvichev et al., 2017). The water flow rate from the deep thermal borehole 3P was estimated at 0.2 to 0.3 L/s (Banks et al., 2011), which allows the average daily yield of methane produced exclusively via hydrogenotrophic methanogenesis to be estimated at 2.26 mL. The isotopic composition (δ^13^C) of CH_4_ was −54.9%o, falling within the range characteristic of a mixture of thermogenic and biogenic methane as described by Ars et al. (2024). The mixing of geological methane with CH_4_ produced by archaea provides a good explanation for its high concentration in the water sample from well 3P. Intensive methanogenesis from dihydrogen may be a probable cause the low H_2_ share in the gas phase. Most likely, hydrogenotrophic methanogenesis in this biotope is limited by the concentration of molecular hydrogen. The observed rate of sulfate reduction may indicate that hydrogenotrophic methanogens are overcompeting sulfate reducers. The low sulfate concentration in the 3P water suggests that sulfate turnover must be rapid.

Molecular hydrogen is an obvious substrate for archaeal methanogenesis in deep subsurface horizons. According to classical concepts of microbial life in the deep subsurface, the production of organic matter by microorganisms is based on chemosynthesis, in which H_2_ and CO_2_ emanating from the Earth’s mantle fuel hydrogenotrophic methanogenesis and sulfate reduction (Nealson et al., 2005). The acronym SLiME (Subsurface Lithoautotrophic Microbial Ecosystems) was proposed by K. Nealson with co-authors to designate geosphere-fueled ecosystems, where electron donors - such as hydrogen and methane - and electron acceptors are supplied entirely by geological processes. A distinctive feature of the West Siberian Basin is its sedimentary nature, associated with the presence of oil shale and vast petroleum reserves. Buried organic matter can serve as a source of organic carbon for heterotrophic prokaryotes in the subsurface within biotopes of this type (Kadnikov et al., 2020). Initial cultivation efforts led to the isolation of a plethora of organotrophic bacteria from the water of well 3P, including polysaccharide-degrading *M. sibirica* (Podosokorskaya et al., 2014), cellulolytic *M. roseus*, (Podosokorskaya et al., 2013a) and *O. apprima* (Podosokorskaya et al., 2013b). The number of uncultivated organotrophic MAGs and cultivated bacteria have been described from samples from two other deep (>2 km) boreholes in the Tomsk region – in the villages of Belii Yar (Kadnikov et al., 2018; Karnachuk et al., 2021) and Chazhemto (Kadnikov et al., 2020). This led to the hypothesis that buried organic matter of marine sediments support a more diverse microbial community than that found in the terrestrial subsurface formed by crystalline bedrocks (Kadnikov et al., 2020). It should be noted that the rate of methanogenesis - or any other microbial process - in water from the Belii Yar and Chazhemto boreholes has not been analysed. Thus, 3P remain the only well in which the activity of methanogenic archaea was quantitively assessed.

Despite the presence of a wide variety of heterotrophic phylotypes, 16S rRNA profiling results showed that the microbial community in water from well 3P was dominated by hydrogenotrophic methanogenic archaea and sulfate-reducing *Bacillota*, which are known for their ability to grow autotrophically using molecular hydrogen. The dominant *Methanothermobacter* was initially characterized by binning the corresponding MAG and subsequently isolated in pure culture. A notable feature of strain K4^T^ is its obligately hydrogenotrophic lifestyle. In the light of the measured rates of methanogenesis and sulfate reduction using radiolabeled substrates, *M. tomskensis* K4^T^ can be considered the primary chemolithotrophic producer of organic matter in the studied biotope.

Few methanogens isolated from the deep subsurface in pure culture have been described in detail. The hydrogenotrophic *Methanobacterium subterraneum* was isolated from groundwater in granitic rock at the Äspö Hard Rock Laboratory, from a depth not exceeding 420 m below sea level (Kotelnikova et al., 1998). An acetoclastic strain of *Methanosarcina hadiensis* was isolated from an underground research facility located within the Boom Clay formation in Belgium at a depth of 161 to 263 m (Giménez et al., 2024). However, sequencing of the V4 region of the 16S rRNA gene from this biotope revealed that the proportion of archaea in the prokaryotic community did not exceed 9.4%, and that in addition to members of *Methanosarcina* representatives of the class *Methanobacteria* were detected (Lipus et al., 2024). The hydrogenotrophic *Methanoculleus horonobensis* strain T10^T^ was isolated from groundwater in an exploratory borehole of the Japan Atomic Energy Agency in Miocene diatomaceous shale at a depth of 362.4–385.7 m below the surface (Shimizu et al., 2013) and the methylotrophic *M. subterranea* strain HC-2^T^ was isolated from the same formation (Shimizu et al., 2015). To the best of our knowledge, *Methanthermobacter tomskensis* is the first thermophilic methanogen isolated from a terrestrial subsurface aquifer at a depth exceeding 500 meters.

## Data Availability

The sequences of 16S rRNA gene fragments, metagenome sequences and annotated sequence of MAG *Methanothermobacter* 3P-20-001 have been deposited in the NCBI GenBank database and are available within the BioProject PRJNA1489495. The GenBank accession number for the 16S rRNA and *mcrA* gene sequences of *M. tomskensis* K4_T_ is OM456264 and PP812523, respectively. The annotated genome sequences of *M. tomskensis* K4_T_ deposited in the NCBI GenBank database under the accession number GCF_022014235.1, the read libraries saved under accession number SRS11832015.
