# Supplementary material for: Archaeal methanogenesis in a deep terrestrial aquifer driven by Methanothermobacter tomskensis sp. nov
Source: Front Microbiol. 2026 Oct 15;17:1973590. doi: 10.3389/fmicb.2026.1973590 (PMC13717691; doi:10.3389/fmicb.2026.1973590)
Supplement: Supplementary file 1 [file Data_sheet_1.docx]

Supplementary Material

Intensive archaeal methanogenesis in a deep terrestrial aquifer driven by *Methanothermobacter tomskensis* sp. nov.

Vladimir E. Trubitsyn^1^, Igor I. Rusanov^2,5^, Viktoria I. Rechkina^1^, Vitaly V. Kadnikov^3^, Daria N. Zasko^4^, Alexey V. Beletsky^3^, Nikolai V. Pimenov^5^, Nikolai V. Ravin^3^, Viktoria A. Shcherbakova^1*^, Olga V. Karnachuk^2^

*** Correspondence:**
Viktoria A. Shcherbakova
vshakola@gmail.com

## 1.1 Supplementary Figure

**Supplementary Figure 1.** Unrooted phylogenetic tree of the genus *Methanothermobacter* showing the position of the new strain, constructed based on mcrA gene nucleotide sequences (467 bp).

The tree was constructed using the Maximum Likelihood algorithm with adaptive bootstrap analysis in the MEGA11 program (Tamura et al, 2021).

Tamura, K., Stecher, G., and Kumar, S. (2021) MEGA11: Molecular evolutionary genetics analysis version 11. *Mol. Biol. Evol.* 38(7), 3022–3027. doi: 10.1093/molbev/msab120

**1.2 Supplementary Tables**

**Supplementary** Table **1.** **Methane production by strain K4^T^ during the growth on different substrates.**

| Substrate | Methane formation*, μmol/ml |
| --- | --- |
| H_2_:CO_2_ (4:1) | 74,5 |
| sodium formate | 0 |
| methanol + H₂ | 0 |
| ethanol + CO_2_ | 0 |
| isobutanol + CO₂ | 0 |
| isopropanol + CO₂ | 0 |
| sodium acetate | 0 |
| acetate + H₂ | 0 |
| methanol | 0 |
| methylamine | 0 |

*Methane formation was measured chromatographically in the gas phase after 14 days of cultivation in the second transfer.

**Supplementary** Table 2**.** **Major methanogenesis genes in the genome of *Methanothermobacter* sp. K4^T^.**

| Protein names | Genes (NCBI numbers) |
| --- | --- |
| Formylmethanofuran dehydrogenase (tungsten-containing isoform) | *fwd*B (L5462_RS04535), *fwd*C (L5462_RS04540), *fwd*A (L5462_RS04545), *fwd*D (L5462_RS04550), *fwd*F (L5462_RS04555), *fwd*E/H (L5462_RS04560) |
| Formylmethanofuran–tetrahydromethanopterin N-formyltransferase | *ftr* (L5462_RS05060) |
| Methenyltetrahydromethanopterin cyclohydrolase | *mch* (L5462_RS06245) |
| Methylene-tetrahydromethanopterin dehydrogenase | *mtd* (L5462_RS05295) |
| 5,10-Methylenetetrahydromethanopterin reductase | *mer* (L5462_RS09170) |
| Tetrahydromethanopterin S-methyltransferase | *mtr*E (L5462_RS05400), *mtr*D (L5462_RS05405), *mtr*C (L5462_RS05410), *mtr*B (L5462_RS05415), *mtr*G (L5462_RS05420), *mtr*H (L5462_RS05425), *mtr*F (L5462_RS05430), *mtr*A (L5462_RS05435) |
| Methyl-coenzyme M reductase | *mcr*B (L5462_RS05370, L5462_RS05395), *mcr*D (L5462_RS05375), *mcr*C (L5462_RS05380), *mcr*G (L5462_RS05385), *mcr*A (L5462_RS05390, L5462_RS05440) |
| Heterodisulfide reductase | *hdr*C (L5462_RS09145), *hdr*B (L5462_RS09150), *hdr*A (L5462_RS05185) |

**Supplementary** Table 3. Gene loci of formate dehydrogenase variants and pyruvate-formate lyase in *Methanothermobacter* genomes.

| Organism (Genome) | Gene loci for protein/enzyme variants | | | |
| --- | --- | --- | --- | --- |
|  | FdhAB (Classical) | FdhABC (Cassini et al., 2023) | FdhFD (This study) | Pfl |
| *Methanothermobacter* sp. K4^T^ (GCF_022014235.1) | L5462_RS02985  L5462_RS02990 | - | L5462_RS01030  L5462_RS01055 | L5462_RS08355 |
| *M. marburgensis* Marburg^T^ (GCF_000145295.1) | MTBMA_RS07430  MTBMA_RS07425 | - | MTBMA_RS00650  MTBMA_RS00675 | - |
| *M. thermautotrophicus* ΔH^T^ (GCF_000008645.1) | MTH_RS05410  MTH_RS05415 | - | MTH_RS07400  MTH_RS07425 | MTH_RS01590 |
| *M. thermautotrophicus* Z-245 (GCF_027555045.1) | ISG36_RS05585  ISG36_RS05580 | ISG36_RS07695  ISG36_RS07700  ISG36_RS07705 | ISG36_RS07560  ISG36_RS07585 | - |
| *M. defluvii* DSM 7466^T^ (GCF_003385755.1) | C7452_RS01190  C7452_RS01195 | C7452_RS06610  C7452_RS06615  C7452_RS06620 | C7452_RS06730  C7452_RS06755 | - |
| *M. tenebrarum* RMAS^T^ (GCF_023167465.1) | - | - | MTTB_RS07990  MTTB_RS07870 | - |
| *M. wolfei* DSM 2970^T^ (GCF_025397995.1) | N5910_RS07730  N5910_RS07735 | N5910_RS03665  N5910_RS03675  N5910_RS03670 | N5910_RS00510  N5910_RS00550 | - |
